# Impact of community and provider-driven social accountability interventions on contraceptive uptake in Ghana and Tanzania

**DOI:** 10.1186/s12939-022-01736-y

**Published:** 2022-09-28

**Authors:** Petrus S. Steyn, Joanna Paula Cordero, Dela Nai, Donat Shamba, Kamil Fuseini, Sigilbert Mrema, Ndema Habib, My Huong Nguyen, James Kiarie

**Affiliations:** 1 UNDP/UNFPA, UNICEF/WHO/World Bank Special Programme of Research, Development and Research Training in Human Reproduction, Avenue Appia 20, 1202 Geneva, Switzerland; 2 Population Council, 204 Yiyiwa Drive, Abelemkpe, Accra, Ghana; 3 Department of Health Systems, Impact Evaluation and Policy, Ifakara Health Institute, P.O.BOX 78373, Dar es Salaam, Tanzania

**Keywords:** Social accountability, Contraception/ family planning, Uptake of contraception

## Abstract

**Background:**

Social accountability, which is defined as a collective process for holding duty bearers and service providers to account for their actions, has shown positive outcomes in addressing the interrelated barriers to quality sexual and reproductive health services. The Community and Provider driven Social Accountability Intervention (CaPSAI) Project contributes to the evidence on the effects of social accountability processes in the context of a family planning and contraceptive programme.

**Methods:**

A quasi-experimental study utilizing an interrupted time series design with a control group (ITS-CG) was conducted to determine the actual number of new users of contraception amongst women 15–49 years old in eight intervention and eight control facilities per country in Ghana and Tanzania. A standardized facility audit questionnaire was used to collect facility data and completed every year in both intervention and control groups in each country from 2018–2020.

**Results:**

In Ghana, the two-segmented Poisson Generalized Estimating Equation (GEE) model demonstrated no statistically significant difference at post-intervention, between the intervention and control facilities, in the level of uptake of contraceptives (excess level) (*p*-value = 0.07) or in the rate of change (excess rate) in uptake (*p*-value = 0.07) after adjusting for baseline differences. Similarly, in Tanzania, there was no statistical difference between intervention and control facilities, in the level of uptake of contraceptives (excess level) (*p*-value = 0.20), with the rate of change in uptake (*p*-value = 0.05) after adjusting for the baseline differences. There was no statistical difference in the level of or rate of change in uptake in the two groups in a sensitivity analysis excluding new users recruited in outreach activities in Tanzania.

**Conclusions:**

The CAPSAI project intervention did not result in a statistically significant increase in uptake of contraceptives as measured by the number of or increase in new users. In evaluating the impact of the intervention on the intermediate outcomes such as self-efficacy among service users, trust and countervailing power among social groups/networks, and responsiveness of service providers, cases of change and process evaluation should be considered.

**Trial registration:**

The CaPSAI Project has been registered at the Australian New Zealand Clinical Trials Registry (ACTRN12619000378123, 11/03/2019).

## Background

Interventions for improving family planning and contraceptive service provision and access can contribute towards the attainment of Sustainable Development Goal 3.7 to ensure universal access to sexual and reproductive health care services, including family planning, information and education, and the integration of reproductive health into national strategies and programmes [1, 2]. Recent estimates show an increase in contraceptive use globally [1, 3]. Low- and middle-income countries (LMIC) have a disproportionately higher unmet need for modern contraception (approximately 218 million women 15–49 years of age), contributing to high rates of unintended pregnancies [1]. Young women aged 15–19 years in LMIC are more likely to have an unmet need and unintended pregnancy. Among 300 million women aged 15–19 years in 2019, 29.8 million currently use contraception, and 15.0 million have an unmet need [4]. Among adolescents, approximately 50% of 21 million pregnancies each year are unintended [1].

The reasons for the high unmet need among women in LMIC vary. They include women's socio-economic status, the lack of access to services, concerns about their health or method side effects, and opposition from a family member [1, 5]. Adolescents face numerous barriers in obtaining and receiving contraceptive care, including fear of exposing that they are sexually active (if they are unmarried) and social pressure to have a child (if they are married) [1]. Provider bias or unwillingness to provide contraceptive care to young unmarried and childless women is another barrier that is often cited [1].

Global guidance and strategies recommend that communities and people directly affected must have the opportunity to be meaningfully engaged in all aspects of family planning and contraceptive programme and policy design, implementation, and monitoring in order to achieve progress towards international development goals and sexual and reproductive health and rights (SRHR) targets [6, 7]. Integrating participation in family planning and contraceptive service provision ensures a rights-based approach to service provision and could better address women's contraceptive needs [6].

Social accountability (SA) is among the participatory processes gaining attention and showing positive outcomes in addressing the numerous and interrelated barriers to reproductive, maternal, newborn, and child and adolescent health (RMNCAH), including family planning and contraceptive service provision [8–10]. Although there is no consensus on the definition of SA in the literature, common elements have been identified [8]. At its core, and for the purpose of this manuscript, SA is defined as citizen-led collective processes for holding duty bearers, including politicians, government officials, and/or service providers, to account for their actions [11].

Positive findings regarding the effect of SA processes on RMNCAH, especially in service delivery and governance outcomes, and some health outcomes are growing ([8] and Appendix). However, specific evidence on SA applied to family planning, and contraceptive service provision remains limited. A 2016 review exploring activities that explicitly engage community members to improve RMNCAH found very little and poor-quality evidence for effects on family planning or birth spacing outcomes [12]. There are recent studies (Appendix) focusing on family planning and contraceptive services that reported positive outcomes, such as improvements in service quality, financial allocation for contraceptive service provision [13, 14], and community awareness [15–17] and participation [13, 14]. Studies have also reported increases in the current use of modern contraceptives [18] and the acceptability of providing family planning to adolescents [19].

A possible reason for the lack of family planning and contraceptive service-related studies could be the belief among programme implementers that family planning is more appropriately addressed through interventions targeting individuals or couples and not through community or public platforms [12].

**Fig. 1:**
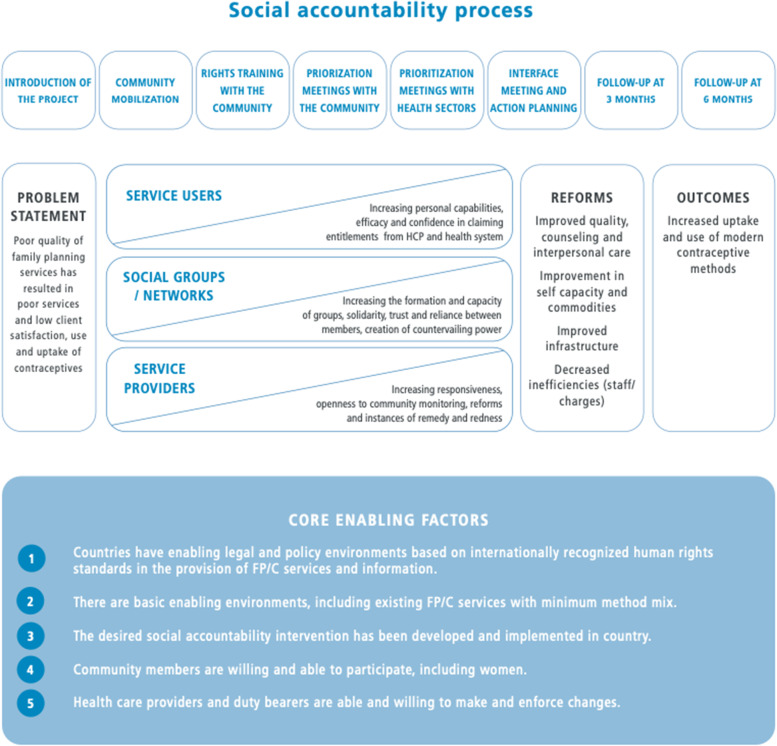
CaPSAI Project Theory of Change – adapted with permission from Steynn 2020 [20]. Image by Little Unicorns

The Community and Provider driven Social Accountability (CaPSAI) Project contributes to the evidence on the effects of SA and participatory processes in the context of family planning and contraceptive programmes. The study was designed according to the Medical Research Council (MRC) guidance on complex interventions based on a theory of change (ToC) (Fig. 1) using a co-designed intervention to account for the complexity of SA processes [20–23]. It accounts for the multiple components required to track the different levels and interrelated outcomes and includes a process evaluation component [20].

The CaPSAI Project ToC (Fig. 1) was developed after a review of the literature and findings from the formative phase research [10, 20, 24]. Firstly, the descriptions, evidence, and programme report from SA interventions applied to health were gathered to understand the common steps being used [9]. Eight steps, which served as a foundation of the intervention, were identified (Fig. 1). The CaPSAI ToC anticipates that engaging community members and health system actors to identify challenges in family planning and contraceptive service provision and develop action plans can lead to improved quality of services, counseling, interpersonal care, staff capacity, and stock management [20]. These, in turn, were expected to facilitate increased uptake and use of modern contraceptive methods by supporting full, free, and informed choice. The CaPSAI ToC is based on several assumptions, including the existence of enabling legal and policy environments for participatory processes and contraceptive use and the ability and willingness of community members and health providers to dialogue on issues related to SRHR.

The study's overall aim is to demonstrate if and how a SA process in the context of family planning and contraceptive programs/services influences quality of care and client satisfaction and whether this results in increased contraceptive uptake and use. Specifically, it aimed to (i) describe and examine how SA processes are implemented and operationalized (understanding behaviours, decision-making processes, and the barriers and facilitators of change, with a view to generalizability); and to (ii) develop more responsive quantitative measures for SA and show the relationship between SA and uptake of contraceptives and use and other family planning behaviours (Table 1).

**Table 1:** Study overview

	**Changes in contraceptive uptake and use**		**Effects of the social accountability process**	
Data Source	**Facility audit**	**Cohort study**	**Cross-sectional survey**	**Process evaluation:** •In-depth interviews (IDI), observations, and document review of intervention steps •Context mapping IDI •Case studies of change IDI and document review
Outcomes	Contraceptive uptake (new users)	Contraceptive use (method discontinuation, continuation and switching)	SA intermediate outcomes (service user and health provider empowerment; expansion of negotiated space)	•Dose, reach and conceptual fidelity •Contextual factors •Reforms or changes resulting from the SA process
Sample	Eight health facilities providing family planning services per group per country	Cohort of 800 women aged 15–49 who are new users of contraception across eight facilities per arm per country	•Two family planning health care providers per facility in the intervention group per country •750 women aged 15–49 who are new and continuing users of contraception in intervention facilities per country	•IDI: Community and district participants and staff at key program/ implementation events; minimum of three interviews in each of eight events at four intervention facilities •Observations: eight events at four intervention facilities •The context mapping interviews were undertaken among community representatives and district-level health actors; Ghana: three IDIs in seven districts = 21 IDIs per time point; Total of 63 IDIs; Tanzania: three IDIs in four districts = 12 IDIs per time point; Total of 35 IDIs (at baseline only 11 IDIs were conducted) •Case studies of change: Ghana—Number of interviews per case: between one and three (20 for nine cases); Tanzania – Number of interviews per case: three (27 for nine cases)

This manuscript will report on the contraceptive uptake outcome measured by the number of new users from a series of facility audits. Other outcomes mentioned in the background section will be reported in separate manuscripts (Table 1).

## Methods

### Intervention

In the context of the CaPSAI study, two civil society organizations (CSO) implementing SA processes that included the eight steps of the ToC were selected from Ghana and Tanzania [21]. To embed the study intervention in local contextual realities CSO partner routines and to account for the processual nature of SA, the study did not define intervention activities prescriptively (Table 2). Instead, the intervention was co-designed with the implementing partners and used conceptual fidelity where the interventions were tracked, comparing the intended activities and how they were implemented [21].

**Table 2:** Eight standard steps of Community and Health Provider driven Social Accountability Intervention (CaPSAI) – reproduced with permission from Steyn 2020 [20]

Step	Description
1. Introduction of the intervention to the community	The implementation partner (a CSO) meets with local leaders, identifies stakeholders, and sets up the infrastructure to deliver the SA intervention
2. Mobilization of participants for the intervention	Community members, service providers, and other health service actors (duty bearers) are gathered by the implementing partner and introduced to the SA process
3. Health, rights, and civic education with community participants	The implementation partner shares information on health awareness and education, and existing service standards. The implementation partner provides training on rights, good governance, and accountability. The group begins to rate existing services against rights-based standards and generate discussion about local priorities
4. Prioritization meeting with community	The implementation partner distills themes and priorities raised by the community. The community groups then collectively score the issues and indicators and set priority areas for action
5. Prioritization meeting with duty bearers	The implementation partner distills themes and priorities raised by the service providers. The providers then collectively score the issues and indicators and set priority areas for action
6. Interface meeting and joint action planning	The implementation partner then holds a joint meeting between the community, the service providers, and health services actors (duty bearers). Following the presentation of results from the prioritization meetings, the community groups and service providers will aim to reach a consensus on the ranking of priority items and the actions required to address them. An action plan with assigned roles and responsibilities will be developed for the following 6- to 12-month period
7. First follow-up meeting with community and duty bearers at three months	Priority areas and action items will be followed up with both the community and service providers. It is at this stage that change is anticipated on the part of health services actors, and remedial actions have taken place, which should be demonstrated in the monitoring activities. For any unresolved issues, these meetings present an opportunity to involve higher-level duty bearers or third-party groups (media/politicians) to increase the pressure to act
8. Second follow-up meeting with community duty bearers at six months	A second follow-up meeting will enable the monitoring of longer-range outcomes and the remedy of unresolved issues raised in the first follow-up meeting. The community and service providers will continue to monitor the action plan for changes in relation to agreed priority areas

### Study design

A quasi-experimental study utilizing an interrupted time series design with a control group (ITS-CG) was conducted to determine the actual number of new users of contraception amongst women 15–49 years old in eight intervention and eight control facilities per country in Ghana and Tanzania during 18 months. A detailed description of the ITS-CG design has been published elsewhere [25].

### Main outcomes

The primary outcome of the study, uptake of modern contraception, is defined as the rate of first-time modern contraception users, expressed as the number of women in the study facilities requesting the use of modern contraception for the first time per month, per 10,000 women of reproductive age, in the catchment area.

### Study setting

Site selection is discussed in detail in a protocol manuscript and the Australian New Zealand Clinical Trials Registry and summarised here [20, 26]. Ghana and Tanzania were selected as the study countries because they had: the presence of an active CSO partner with local experience in delivering a SA intervention with the eight steps referred to above (Table 2); low modern contraceptive prevalence (mCPR) rate, excluding barrier contraception; availability of contraceptive services to the client at no cost or where cost was not a barrier to access. Other criteria included an enabling environment for the health system to act on SA activities and the existence of established structures to link the community with the health system [20, 26].

In Ghana, mCPR increased from 18.7% in 2003 [27] to 22.2% in 2014 and to 25.0% in 2017 [28] among currently married women. Among all women, it increased from 15.3% in 2003 [27] to 18.2 in 2014 [29] and then to 19.5 in 2017 [28]. Unmet need, marginally decreased from 34.0% in 2003 [27] to 29.9% in 2014 [29] and increased to 33.6% in 2017 [30].

The Ghana health system structure includes three main levels of health service provision: primary (health centres, clinics, and Community-based Health Planning and Services, known as CHPS), secondary (district and regional hospitals), and tertiary (teaching hospitals, with the Ministry of Health as the overarching body overseeing the health system and the Ghana Health Service as the implementing branch). Family planning and contraceptive services are provided at all levels of health service provision.

In Tanzania, the mCPR rate is at 28.9% despite a steady increase over the past decade, from 18% in 2004 [31]. Among currently married women, injectables (8.5%), pills (5.1%), and male condoms (4.2%) are the most used. Contraceptive use varies according to background characteristics, with married women in urban areas being more likely to use a modern method than rural women. The government or the public sector is the main source of modern methods (65% of users) [31, 32].

The Tanzanian health service delivery is categorized into three levels: primary care, secondary, and tertiary care. The primary care level consists of dispensaries that only provide outpatient care and refer complicated cases to the secondary care level. The secondary level consists of health centres and district hospitals that provide both outpatient and inpatient care. All complicated cases are referred to the tertiary level. The tertiary level includes regional referral hospitals and super-specialized hospitals. Family planning and contraceptive services are provided in all of the facilities at all levels.

Districts selected had no SA intervention in family planning and contraceptive programmes at baseline and sufficient health facilities offering family planning services. Data for at least 20 health facilities in the selected districts were used to select eight intervention and eight control facilities. Selected facilities had at least a barrier method, a short and long-acting method, emergency contraception, and referral for permanent methods available. The matching of study and control sites was done on the type and level of facilities, the average number of service users, and the number of new users. Facilities with the following characteristics were excluded: (i) a mean of less than 50 new users per month, (ii) private and NGO facilities, and (iii) tertiary or secondary and referral facilities.

The interventions were conducted in the intervention facility catchment areas, targeting community members, health professionals, and other duty bearers. Study outcomes were measured at the facility level, where we expected changes in uptake.

### Sample size and statistical analysis

The detailed estimation of study sample size and power were done in two ways and described in Habib et al. [25] and Steyn et al. [20]. The facility was the unit of measurement used for Poisson regression, and the monthly data points were the unit of measurement for time series regression. At pre-intervention (or baseline) and at post-intervention, different sample size estimates were computed using a two-sided t-test with Type I error at 5% level, statistical power at 80%, and assuming equal variance [25]. These various sample size estimates provided assumed a constant denominator and a pooled variance for the mean number of new users at pre-intervention in both groups of between 100 and 200 new users per facility, per month, and a difference in uptake per facility per month, at post-intervention of between 60 and 200 new users. From this, eight facilities per intervention or control group were chosen to detect about a two-fold increase in the rate of new users with 80% power and allowing for a 5% Type I error.

Using the monthly data points as the unit of analysis, we relied on the simulation-based power calculation provided by Zhang et al., 2011 taking effect size and time periods into account [33]. Assuming an effect size of 2.0 (derived from 100 new users in the control group and 200 new users in the intervention group and a pooled standard deviation of 50) and autocorrelation of up to 0.3, we would need a total of 12 data points. This is equivalent to six monthly pre-intervention data points and six-monthly post-intervention data points to achieve 80% power at a p = 0.05 statistical significance level [33].

As the facility was used as the unit of analysis, all potential confounders at the facility level were recorded. An ITS segmented Poisson regression model was used to estimate both the level changes in first-use of modern contraception rate and changes in time trends for the rate of modern contraception first-use rates per 10,000 women-months, after the introduction of the intervention. The generalized estimating equation (GEE) Poisson segmented regression model allowed for adjustment for correlation due to repeated observations from the same facilities while also adjusting for important baseline facility characteristics [20, 25, 26].

### Data collection

A standardized questionnaire [34] was used for the facility audit and completed on a yearly basis in both control and intervention sites. This was done through interviews with facility staff, preferably a manager. The audit was adapted and shortened from the Service Availability and Readiness Assessment (SARA) tool [35]. The service statistics were continuously recorded, and the number of new users was determined by the facility staff and recorded monthly to minimize selection/performance bias. The research staff was trained to avoid misinterpretation of data and ensure that the outcome was assessed in a consistent manner. Data were double entered into a web-based *OpenClinica®* database and adhered to principles of data management as specified in the data management standard operating procedure [36].

### Enrolment

**Fig. 2:**
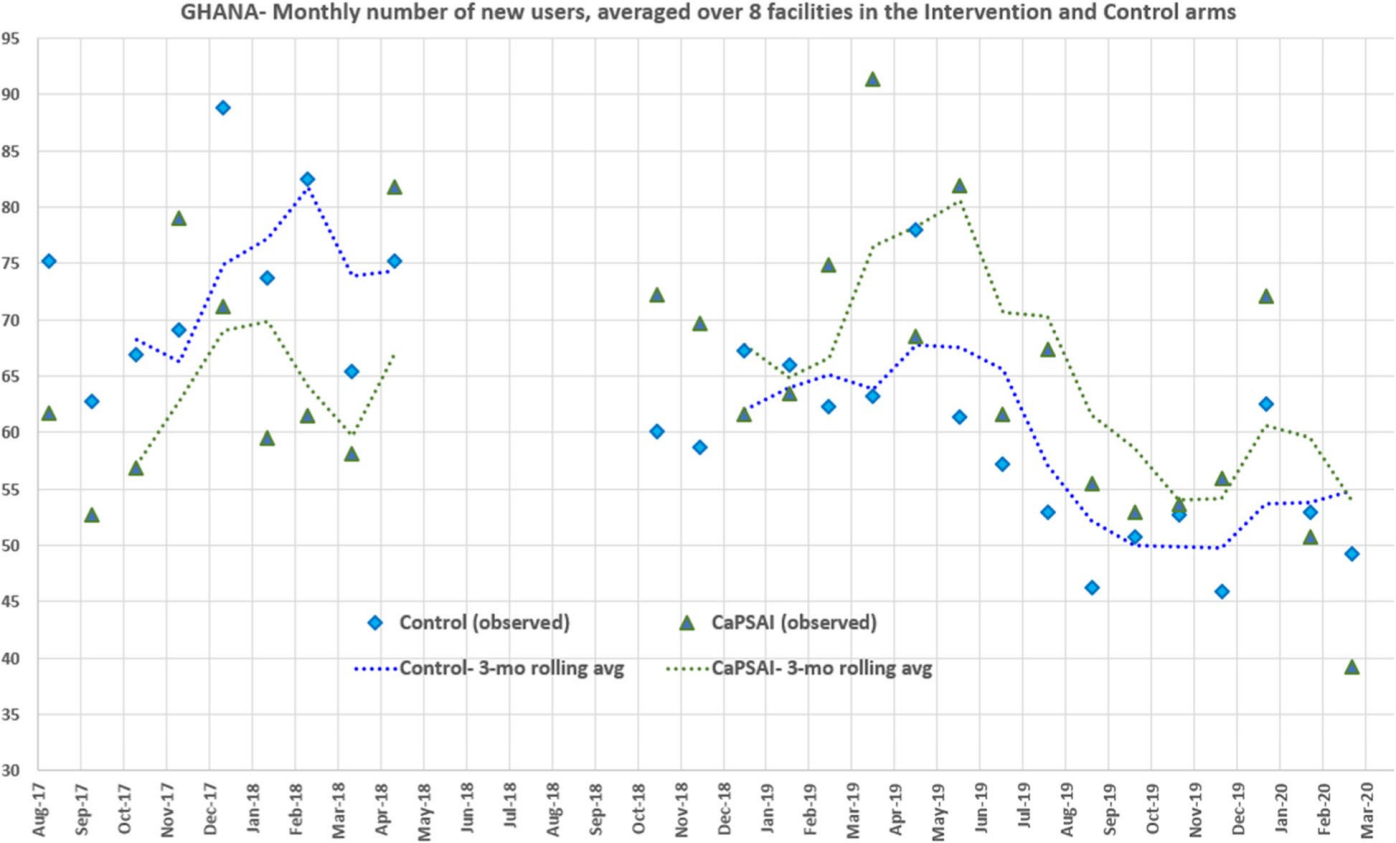
Intervention and Control Facilities: Averaged contraceptive new user count over study time, by study group, Ghana

**Fig. 3:**
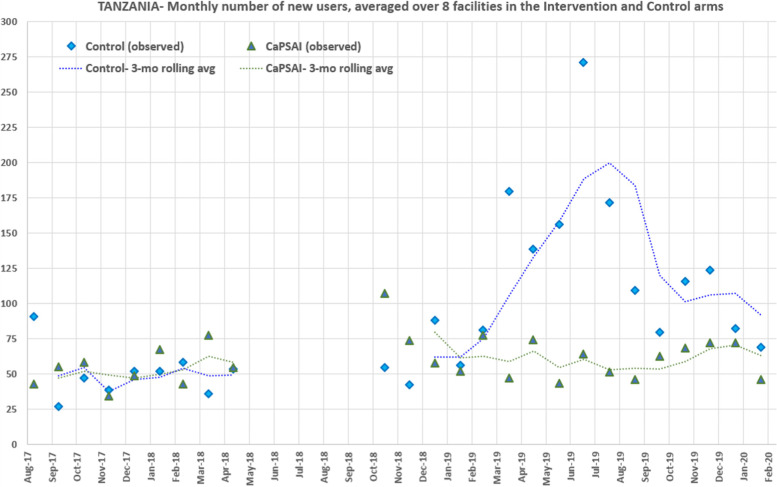
Intervention and Control Facilities: Averaged contraceptive new user count over study time, by study group, Tanzania

The service statistics, which included the monthly number of new users, were collected in the eight study and eight control sites in the two countries from the facility staff and documentation. This was done for six months before the intervention to determine the baseline (March to April 2018), interim (March to April 2019), and at end line (March to April 2020). Adaptations to the data collection had to be made during the COVID-19 pandemic, which restricted in-person data gathering. This caused a delay in data gathering until May 2020 in Ghana. In Ghana, 31 months of data were collected from September 2017 to March 2020. In Tanzania, 31 months of data were collected from August 2017 to February 2020. The number of new contraceptive users per month, per study group, averaged across facilities over the study time is shown in Fig. 2 and Fig. 3 for Ghana and Tanzania, respectively.

## Results

### Facility characteristics at baseline for both countries

The facility characteristics at baseline for the respective countries are presented in Table 3. In Ghana, the type of facilities included in the control group were one district hospital, five health centres, one Community-based Health Planning and Services (CHPS), and one reproductive and child health clinic as compared to one district hospital, six health centres, one CHPS in the intervention group. In Tanzania, the type of facilities included in the control group were four health centres or clinics, four dispensaries against two health centres or clinics, and six dispensaries in the intervention group. In both countries, the managing authority for all facilities was the public health authority. All facilities have family planning and contraceptive services, which are offered for four to eight hours per day. In Ghana, contraceptive methods are free, but the services are not. In Tanzania, both contraceptive methods and services are free. Staff in all facilities in Ghana received specified family training during the previous two years, whereas seven facilities in the control group and five facilities in the intervention group in Tanzania received training in the previous two years. All intervention and control facilities in both countries met the inclusion criteria for the minimum types of methods provided. Most (15 of 16) facilities in both countries conducted various outreach programmes, including maternal, child, and newborn health care, in addition to family planning.

**Table 3:** General description of facilities in Ghana and Tanzania at baseline

Characteristics	Ghana		Tanzania	
	**Control** **(*n* = 8)**	**Intervention (*n* = 8)**	**Control** **(*n* = 8)**	**Intervention** **(*n* = 8)**
**Type of facilities**				
2- District Hospital	**1**	**1**	**0**	**0**
3- Health Centre /Clinic	**5**	**6**	**4**	**2**
4- Health Post (in Ghana, Community-based Health Planning and Services- CHPS)	**1**	**1**	**NA**	**NA**
6- Dispensary (Tanzania)	**NA**	**NA**	**4**	**6**
7- Maternal/ Child Health Clinic	**1**	**0**	**0**	**0**
**Managing Authority**				
Government/ Public	**8**	**8**	**8**	**8**
NGO/ Not-For-Profit	**0**	**0**	**0**	**0**
**Number of facilities that have FP services open 4–8 h per day**	**8**	**8**	**8**	**8**
**Number of facilities providing FP service for free**	**0**^a^	**0**^a^	**7**	**8**
**Number of facilities that have received FP training in the last two years**	**8**	**8**	**7**	**5**
**Have an outreach programme**	**7**	**8**	**8**	**7**

^a^In Ghana, contraceptive methods are free, but services are not

### Uptake in Ghana

**Fig. 4:**
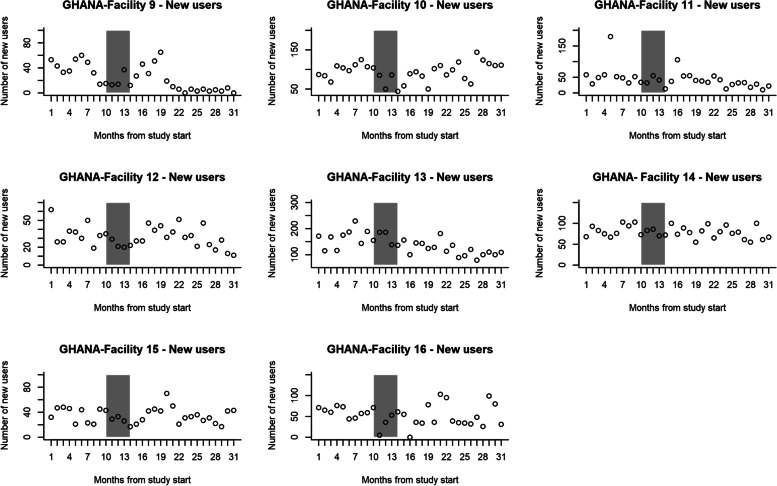
The number of first-time contraceptive users per Control facility per month, Ghana

**Fig. 5:**
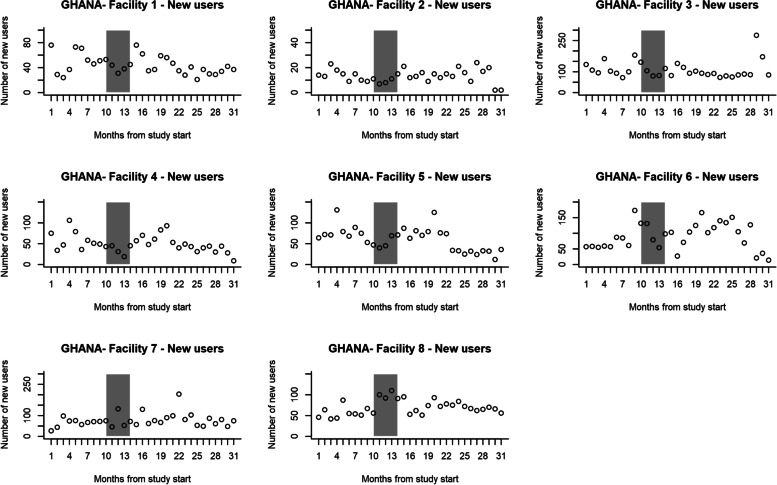
The number of first-time contraceptive users per Intervention facility per month, Ghana

In the control group, the number of first-time contraceptive users nine months before the intervention was 5278 and 7901 in the 17 months post-intervention. In the intervention group, it was 4659 at pre-intervention and 8743 at post-intervention. The numbers of first-time contraceptive users per month are shown in Fig. 4 (control group) and Fig. 5 (intervention group). Table 4 shows the two-segmented Poisson GEE model log uptake parameter estimates using all available 26 data points (9 pre- and 17 post-intervention). The model excludes the intervention roll-out phase of five months. For the control group, the pre-intervention rate of change in uptake was not significantly different from zero (*p*-value = 0.8). Post-intervention, the level and rate of change in uptake (*p*-value = 0.9) and (*p*-value = 0.2), respectively, were not significantly different from pre-intervention in the control group. When the pre-intervention study- and control groups were compared, the uptake levels and rate of change in uptake were not significantly different between the intervention (*p*-value = 0.8) and control facilities (*p*-value = 0.2). Post-intervention, the uptake level rate of change in uptake was not statistically significant different between intervention (*p*-value = 0.07); and control groups (*p*-value = 0.07) after adjusting for baseline differences.

**Table 4:** GHANA—Two-segmented Poisson GEE Model Log(Uptake) parameter estimates (AR[1] working correlation) using all available 26 datapoints (9 pre-, 17 post) (excluding intervention roll-out phase)

Control Arm						Intervention Arm					
**Parameter description**	**Parameter estimate** (Log Uptake) (αi)	**SE**	**95%CI**		***p***>**-value**	**Parameter description**	**Parameter estimate** (Log Uptake) (αi)	**SE**	**95%CI**		***p*-value**
			**LL**	**UL**					**LL**	**UL**	
**Pre-Intervention phase (Pre-Step 1)**						**Pre-Intervention phase (Pre-Step 1)**					
** Baseline Level, (**α0)	4.777	0.206	4.374	5.181	< 0.0001^*^	** Excess level, (**α4) *(vs Control)*	-0.088	0.350	-0.774	0.599	0.80^**^
** Trend** (α1)	0.003	0.013	-0.022	0.028	0.83^*^	** Excess trend** (α5) *(vs Control)*	0.038	0.030	-0.020	0.096	0.20^**^
**Post-Intervention phase (Post Step 6)**						**Post-Intervention phase (Post Step 6)**					
** Excess level** (α2) *( vs control grp pre-intervention baseline level)*	-0.023	0.214	-0.442	0.395	0.91	** Excess level** (α6) *( vs Control)*	0.657	0.368	-0.065	1.378	0.074^¥^
** Excess trend** (α3) *(vs control grp pre-intervention trend)*	-0.015	0.013	-0.040	0.010	0.23	** Excess trend** (α7) (vs control)	-0.056	0.031	-0.116	0.0047	0.071^¥^

^*^ *p*-value < 0.05 indicates that uptake baseline levels and/or trends during pre-intervention phase, for the control facilities, is significantly different from zero

*p*-value < 0.05 indicates that the post-intervention phase uptake level and/or trend, for the control facilities, is statistically significant (vs pre-intervention phase)

^**^ *p*-value < 0.05 indicates that the pre-intervention uptake level and/or trend, for the CaPSAI facilities is significantly different from the Control group facilities) (i.e. excess pre-intervention change ( is significant)

¥ *p*-value < 0.05 indicates that excess uptake level and/or trend in Intervention arm during post intervention phase is statistically significantly different from the excess change in the Control arm (The *p*-value tests the overall effectiveness of the CaPSAI on changing the level and/or trend in contraceptives uptake, after all known pre-intervention and other confounding factors in both groups have been accounted for

### Uptake in Tanzania

**Fig. 6:**
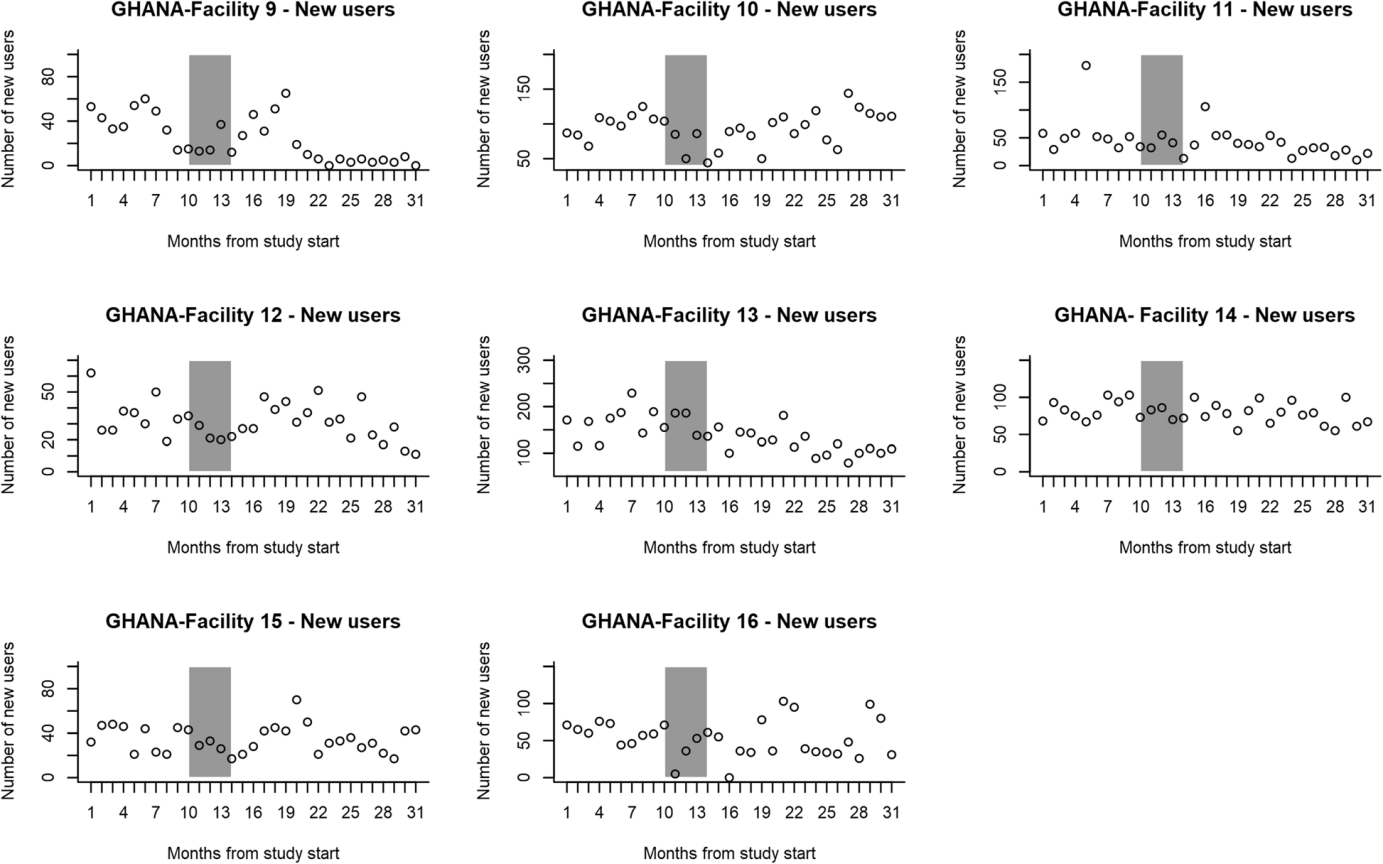
The number of first-time contraceptive users per Control facility per month, Tanzania

**Fig. 7:**
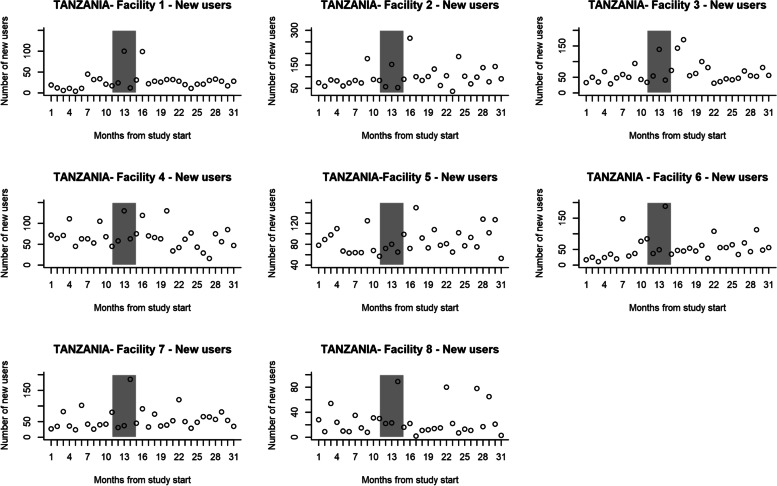
The number of first-time contraceptive users per Intervention facility per month, Tanzania

In the control group, the number of first-time contraceptive users ten months before the intervention phase was 3454 and 13566 in the 16 months post-intervention. In the intervention group, it was 3766 in pre-intervention and 8138 in post-intervention. When outreach services were excluded from the control group, the number of first-time contraceptive users was 2180 at pre- and 6182 at post-intervention. In the control group, there were 3766 and 6511 first-time contraceptive users, respectively. The number of first-time contraceptive users per month is shown in Fig. 6 (Control group) and Fig. 7 (intervention group). Table 5 shows the two-segmented Poisson GEE model log uptake parameter estimates using 26 data points (10 at pre- and 16 at post-intervention). The model excludes the intervention roll-out phase of five months. For the control group, the pre-intervention rate of change in uptake was not significantly different from zero (*p*-value = 0.34). Post-intervention the uptake (*p*-value = 0.021) and (*p*-value = 0.94) respectively were not significantly different from pre-intervention in the control group. When the pre-intervention study and control groups were compared, the uptake levels were significantly lower than in the control group facilities (*p*-value = 0.016). However, the pre-intervention rate of change was not significantly different *p*-value = 0.66). Post-intervention, the difference in uptake level and rate of change in uptake were not statistically significant between the intervention(*p*-value = 0.053) and control groups (*p*-value = 0.053) after adjusting for baseline difference.

**Table 5:** Tanzania—Two-segmented Poisson GEE Model Log(Uptake) parameter estimates (AR(1) working correlation) using all available 26 datapoints (9 pre-, 17 post) (excluding intervention roll-out phase)

Control Arm						Intervention Arm					
**Parameter description**	**Parameter estimate** (Log Uptake) (αi)	**SE**	**95%CI**		***p*-value**	**Parameter description**	**Parameter estimate** (Log Uptake) (αi)	**SE**	**95%CI**		*p***-value**
			**LL**	**UL**					**LL**	**UL**	
**Pre-Intervention phase (Pre-Step 1)**						**Pre-Intervention phase (Pre-Step 1)**					
** Baseline Level, (**α0)	4.858	0.214	4.440	5.277	< 0.0001^*^	** Excess level, (**α4) *(vs Control)*	-0.610	0.254	-1.108	-0.112	0.016^**^
** Trend** (α1)	0.0176	0.018	-0.018	0.054	0.34^*^	** Excess trend** (α5) *(vs Control)*	0.011	0.025	-0.038	0.060	0.66^**^
**Post-Intervention phase (Post Step 6)**						**Post-Intervention phase (Post Step 6)**					
** Excess level** (α2) *( vs control grp pre-intervention baseline level)*	0.472	0.154	0.171	0.773	0.021	** Excess level** (α6) *( vs Control)*	0.388	0.302	-0.203	0.980	0.198^¥^
** Excess trend** (α3) *(vs control grp pre-intervention trend)*	0.0016	0.019	-0.037	0.040	0.94	** Excess trend** (α7) (vs control)	-0.053	0.027	-0.107	0.0003	0.053^¥^

^*^p-value < 0.05 indicates that uptake baseline levels and/or trends during pre-intervention phase, for the control facilities, is significantly different from zero

*p*-value < 0.05 indicates that the post-intervention phase uptake level and/or trend, for the control facilities, is statistically significant (vs pre-intervention phase)

^**^p-value < 0.05 indicates that the pre-intervention uptake level and/or trend, for the CaPSAI facilities is significantly different from the Control group facilities) (i.e. excess pre-intervention change ( is significant)

^¥^*p*-value < 0.05 indicates that excess uptake level and/or trend in Intervention arm during post intervention phase is statistically significantly different from the excess change in the Control arm (The *p*-value tests the overall effectiveness of the CaPSAI on changing the level and/or trend in contraceptives uptake, after all known pre-intervention and other confounding factors in both groups have been accounted for

### Sensitivity analysis

**Fig. 8:**
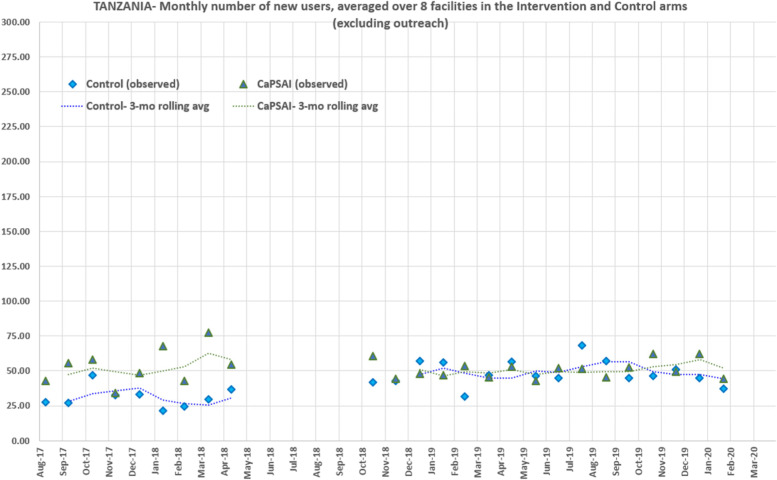
Intervention and Control Facilities: Averaged contraceptive new user count over study time, by study group, Tanzania – Excluding outreach activities

Two sensitivity analyses were done. Firstly, in Tanzania, outreach activities by non-governmental organizations were more frequent during specific months in the control facilities. New users from these outreach activities were added to the facilities' monthly data, although service provision, including the provision of commodities, was not done by the facility staff. Figure 8 shows the crude number of new users per month (excluding the outreach services) averaged over each control and intervention facility. In the GEE model, the exclusion of new users enrolled in outreach activities in intervention and control sites did not significantly change estimates of intervention effect (results not shown).

A second sensitivity analysis was done in both countries, including data from six months after the intervention during the follow-up meetings to monitor, support, and strengthen the identified prioritized actions. In Ghana, this did not significantly change estimates of the intervention effect. There were no significant changes in Tanzania when the outreach data were excluded (results not shown).

## Discussion

The study's overall aim is to demonstrate how a SA process in family planning and contraceptive programmes and services influences the quality of care and client satisfaction and whether this leads to increased contraceptive uptake and use. We report here on the relationship between SA and uptake of modern contraceptives.

In this ITS-CG, we did not find a statistically significant increase in uptake of contraceptives in the intervention compared to the control facilities as measured by the number of new users before and after the intervention. This finding is consistent with other recent studies, including two large-scale, randomized controlled trials that evaluated health outcomes in SA. The Accountability Can Transform Health (ACT Health) scaled up the Power to the People [37] study and found no statistically significant effects on utilization rates and robust null effects on health outcomes, including child mortality [38]. However, some positive impacts on treatment quality and patient satisfaction were identified [38]. The Transparency for Development study reported that a community-led transparency and accountability programme to improve maternal and newborn health did not have a statistically significant impact on the use or content of the services, nor on perceptions of civic efficacy or civic participation among recent mothers in the communities where it was offered [39].

Several reasons have been postulated to explain the lack of evidence on health outcomes and outcomes specific to contraceptive service provision. Methodological challenges have been identified in researching SA processes as it varies from context to context and entails multiple and interrelated components, steps and actors and several simultaneous behaviour change processes [40]. Quantitative or clinical study designs that reduce or attempt to control the complexity of SA may lead to several dimensions, such as political factors and power dynamics, being missed [41]. This emphasizes the importance of applying a complex designed methodology to account for the multiple components, including a process evaluation with context evaluation to track the different levels and interrelated outcomes of SA [22, 40].

### Strengths of the study

We used a quasi-experimental design with a control group as randomization was not feasible in this project. A control group is essential to ascertain that the reported findings are attributable to the intervention. An ITS-CG design was used in this quasi-experimental design to evaluate the effectiveness of the intervention to show the accelerating uptake of modern contraceptives [25]. The strength of this segmented ITS modeling is that it can demonstrate the effect of an intervention on the number or the trend in uptake of modern contraceptives. ITS-design accounts for time and allows non-parallel trends between the two groups [42]. It also addresses varying durations of intervention roll-out and delayed effects of the intervention, which are characteristic of SA processes [25, 43]. Other advantages include minimizing a history bias by allowing for both the before-after intervention comparison and the intervention-control group comparison to be made [44].

The contraceptive uptake outcome reported here should be interpreted with other CaPSAI outcomes, such as the intermediate outcomes, case studies of change, and the process evaluation, including context mapping – these will be reported in detail elsewhere.

The CaPSAI Project followed the MRC guidance of studying complex interventions and incorporated SA intermediate outcome measures and an extensive process evaluation [20]. Triangulating the process evaluation findings, intermediate outcome measures, and the health outcomes evaluated by CaPSAI could allow us to gain a fuller picture and unpack the causal pathways. Intermediate outcomes of SA and the changes at the facility level, as depicted by case studies of change, were shown to affect service delivery and at the community level in the CaPSAI Project. Previous studies have shown positive results on these levels of outcomes in both RMNCAH and contraceptive service provision (Appendix). CaPSAI validated psychometric scales were used to measure SA outcomes such as women's^1^ acknowledgment and awareness of rights, empowerment and self-efficacy, and participation in collective action [20, 45]. Throughout the study, the researchers also tracked the stories of change which are transformations at the facility level directly stemming from the prioritizations and joint action planning done as part of the intervention [20]. Selected case studies were verified through document review and IDI with key informants. These case studies provide a better understanding of how the changes occurred and describe the context and factors that enabled them.

The process evaluation's main aim was to document the implementation of the intervention and understand whether this was done as intended and whether it reached the target audiences. It also documented the facilitators and barriers to the intervention [20]. In a previously published process evaluation of a community monitoring programme initiated by a national health body, the authors were able to evaluate the quality of activities conducted, the level of community participation and engagement with health providers, and identify the gaps in the implementation [46].

The process evaluation also included context mapping, which was conducted at baseline and on a yearly basis throughout the study period (Steyn 2020). Specifically, the objectives were to gauge the types of family planning promotion done at the community level and whether there were participatory activities done that may have affected the intervention or the rates of contraceptive uptake in both the intervention and control groups, and how these changed over time. Understanding how SA initiatives interact or are influenced by contextual factors has been underlined in the existing literature. Examining the different types of actors, political or civil society systems, and the relationships between them can support the development of context-sensitive theories of change when adapting or replicating SA to different contexts [47] and identify and account for the enabling environment and constraints for SA [48]. SA exists in complex ecosystems that may include other accountability mechanisms that may have implications on how a SA process functions and is perceived [49]. Contextual factors and influences have shown how the synergistic influences of the individual, spousal, organizational, and societal factors that influence individual preferences but also constrain how individuals implement them [50].

### Country context

The findings of the context mapping conducted in the intervention and control districts in both Ghana and Tanzania will be reported elsewhere. Here, we report the contextual factors identified in published literature and other studies on participatory processes in the two settings. Various family planning interventions have been implemented in Ghana over the years (e.g., [51, 52]). However, nationally representative data from 2017 indicate that since 2003, the mCPR rate among married women has increased by a mere six percentage points to 25%, and unmet need has remained at an average of 30% over the same period [28]. Specific to Central region, mCPR increased from 13% in 2003 [27] to 28% in 2014 [29] and declined to 25% in 2017 [28]. Unmet need for contraception in the region decreased from 50% in 2003 [27] to 29% in 2014 [29]. Reasons for use and non-use continue to be studied and vary depending on context. Place of residence, educational level [53, 54], marital status, partner consent and support, religious beliefs [55], and age [56] have all been identified as determinants of contraceptive use. At the same time, fear of side effects, concern with the menstrual irregularities caused by hormonal methods, myths, and misconceptions have been cited as reasons for non-use [56, 57]. An evaluation of a personalized, community-based counseling and referral programme on modern contraceptive use in selected urban areas of Ghana found that the intervention did not achieve its aim to reach all reproductive-aged women in the community, and there was no significant effect of the intervention at either programme close or two years later [52]. Another study on the long-term impact of the Navrongo project in Northern Ghana, which included social mobilization activities, found a significant fertility decline arose in the project's early years [51].

The finding on uptake could be explained by the steady but slow increase in the use of modern contraceptives in Tanzania regardless the number of nationally implemented efforts which are nationally implemented. A recent study [58], conducted in Tanzania found that the overall use of modern contraceptives has increased only by 11.3 percent, that is 20.0% in 2004/2005 to 32% in 2015/2016. The national target of 45% mCPR by 2020 was not achieved [58]. An example of national-level efforts made includes the publication of the National Family Planning Costed Implementation Program 2010–2015, which targeted a national CPR of 60% by 2015. The National Family Planning Research Agenda was also updated to identify gaps in family planning through evidence-based knowledge. The One Plan II was launched, aiming to achieve a national mCPR of 45%, reduce the unmet need of contraception to 10% by 2020 and double the number of family planning users to 4.3 million by 2020 as part of family planning 2020 initiatives. The use of modern contraceptives in Tanzania is not uniform. It varies due to individual and regional determinants. A study [59] conducted in Tanzania proposed that contraceptive use increases with household wealth and with an increasing number of children ever born. Further, it was identified that in Tanzania, women who live in urban areas and who see community-based distribution workers and clinic staff as the primary people to talk about family planning are more likely than other women to use contraceptives. Education level has been identified as an important factor influencing contraceptive use and may vary at the regional level [59–62].

### Limitations of the study

The CaPSAI Social Accountability intervention was delivered in contexts where many other interventions were happening in both groups. Specific outreach programmes (e.g., awareness campaigns and specific days to insert subcutaneous implants, policy, and political changes) may have affected community or provider behaviour towards contraception and contraceptive use. History bias occurred as non-study interventions and other activities affecting modern contraceptive uptake were implemented during the roll-out of the study intervention. In Tanzania, we identified that outreach activities were being included in facility data. This could have affected our facility selection process as one of the selection criteria was that facilities had to have at least 50 new users per month on average. Ongoing outreach activities are captured and will be reported elsewhere. The existence of multiple programmes to improve family planning and contraceptive service provision can lead to synergies resulting in the integration of activities [13]. However, these need to be considered when designing programmes and evaluating the impact.

A limitation of ITS-CG is that it does not account for the lack of intervention coverage and depends on whether the intervention is rolled-out to the facilities and respective catchment communities. Another limitation is dose–response, defined as the degree of completeness of the reforms deemed necessary by the community at the end of step 6. The degree of completeness and quality of reforms may influence community satisfaction and uptake of modern contraception when the post-intervention assessment was done. The models cannot account for this dose–response scenario nor the variation in satisfaction levels between communities receiving the intervention. The case studies of change conducted as part of CaPSAI will shed some light on whether prioritized issues have been addressed and in what quality. Seasonal autocorrelation could also not be accounted for in the model for the CaPSAI study since it requires minimum of 24 monthly data points, distributed on the same calendar month at pre- and post-intervention [43].

Study designs using longer time series observations have been shown to be reliable in estimating the effect of the interventions and can robustly model the underlying trends, changes, and random fluctuations [25, 43, 63]. In CaPSAI, we used a shorter time series design with ten pre-intervention and 18 post-intervention observations. Shorter time-series designs are still being used as they are considered more feasible, less resource-intensive than more extended time series, and are used primarily when the interest is in the assessment of the effectiveness of an intervention during a limited period [25]. Multiple rounds of the intervention and a longer follow-up may have shown statistical significance.

A control group for the ITS-CG design must be chosen carefully [42] to ensure baseline comparability to the intervention group. Efforts were made to achieve covariate balance at baseline by considering key characteristics of facilities and catchment areas (rural and urban, level of facility, average number of new users per facility). However, there may have been key differences that could not be accounted for during site selection.

## Conclusion

The data did not show a statistically significant increase in uptake of contraceptives using new users as an indicator for an intervention of six months. In evaluating the impact of the intervention results on intermediate outcomes such as self-efficacy among service users, trust and countervailing power among social groups/networks, and responsiveness of service providers, cases of change and process evaluation should be considered.

This SA intervention was delivered in the context of numerous other interventions addressing the same outcomes that may affect the study's power, i.e., specific outreach programmes. Process evaluation and reach of these programmes are important to contextualize the results.

There is a need for further research on the impact of SA, including evaluating changes in attitudes, behaviour, and experiences of providers, users, and social groups. Experimental designs to understand potential links between SA and service utilization should include counterfactual analysis and be supported by structured process evaluation.

## Data Availability

The de-identified dataset used and/or analysed during the current study can be requested from the Primary Sponsor or Principal Investigators, and data will be shared contingent on approval by the internal review and approval by local internal ethics review board.

## Appendix

Table 6.

**Table 6:** Reported outcomes related to RMNCAH and family planning and contraceptive service provision

Outcome area	Reported outcomes related to RMNCAH	Reported outcomes specific to family planning and contraceptive service provision
Governance	•Increased Participation [64–66], Increased Transparency [65, 67] •Increased confidence among women to claim rights and make demands [15, 68, 69] •Increased community engagement in decision-making [65]	•Increased participation on FP issues, including among religious and cultural leaders [13]
Health financing	•Increased resources [16, 70, 71]	•Increased resourced dedicated specifically for FP [13, 14]
Service Delivery	•Available medical equipment [37, 65] and supplies [37, 72, 73] •Improved Infrastructure [65]; rebuilding and re-opening of non-functional centers [16] •Reduction in waiting time [73] •Increased home visits to identify pregnancies [74] and during pregnancy [75]	•Cleaner facilities [13] •Removal of informal fees [13]
Service Providers	•Increased staffing [14, 65] •Less absenteeism [37, 73] •Improved morale [70] •Improved training and supervision [16, 70, 74] •More engaged provider–client interaction [65, 75] •Improved awareness of community needs [15, 76]	•More engaged provider–client interaction on FP [13] •Improved awareness of community FP needs [14]
Knowledge & information	•Safe Sex/high risk behaviour [77, 78] •Knowledge of health issues, including MNH [15, 16] Knowledge of health rights and entitlements [68, 79] •Male involvement in MNH [66] •Awareness of community groups that engaged with health system [75]	•Knowledge of health issues, including FP [15, 16] •Increased willingness to dialogue in public on FP [14] and SRHR problems faced by adolescents [17]
Service utilization	•Increased utilization of MNCH services [15, 19, 37, 72] Improved access to services [18] •Use of skilled birth attendants [37] •Increased satisfaction [75]	•Increased current use of FP [75]
Health outcomes	•Child weight [37] •Maternal Mortality Ratio [80]	

## Abbreviations

CaPSAI: Community and Provider driven Social Accountability Intervention
CSO: Civil Society Organizations
GEE: Generalized Estimating Equation
IDI: In-depth interviews
ITS-CG: Interrupted time series design with a control group
LMIC: Low- and middle-income countries
MRC: Medical Research Council
mCPR: Modern contraceptive prevalence rate
RMNCAH: Reproductive, maternal, newborn and child, and adolescent health
SA: Social accountability
SRHR: Sexual and Reproductive Health and Rights
ToC: Theory of change

## Footnotes

1 A health provider survey to measure social accountability intermediate outcomes was also used in CaPSAI but as the psychometric scales were not validated, we will not be reporting on the results.
